# Biological Status and Dietary Intakes of Iron, Zinc and Vitamin A among Women and Preschool Children in Rural Burkina Faso

**DOI:** 10.1371/journal.pone.0146810

**Published:** 2016-03-18

**Authors:** Yves Martin-Prevel, Pauline Allemand, Laetitia Nikiema, Kossiwavi A. Ayassou, Henri Gautier Ouedraogo, Mourad Moursi, Fabiana F. De Moura

**Affiliations:** 1 Institut de Recherche pour le Développement, Research Unit 204 ‘Nutripass’, Montpellier, France; 2 Institut de Recherche en Sciences de la Santé, Ouagadougou, Burkina Faso; 3 HarvestPlus, International Food Policy Research Institute, Washington, DC, United States of America; TNO, NETHERLANDS

## Abstract

**Background:**

Food-based approaches such as biofortification are meant to sustainably address micronutrient deficiencies in poor settings. Knowing more about micronutrient intakes and deficiencies is a prerequisite to designing and evaluating interventions.

**Objective:**

The objectives of the study were to assess biological status and dietary intakes of iron, zinc and vitamin A among women and children aged 36–59 months in rural Burkina Faso and to study relationships between intake and status to better inform future food-based interventions.

**Design:**

A cross-sectional survey was carried out in two rural provinces of Burkina Faso on a random cluster sample of 480 mother-child pairs. Dietary data was obtained by 24-hour recalls repeated on a random sub-selection of 37.5% of subjects to allow calculation of nutrient’s probability of adequacy (PA). Biomarkers were measured on a sub-sample of 180 mother-child pairs. Blood samples were analyzed for hemoglobin, serum ferritin, soluble transferrin receptors (sTfR), C-reactive protein, alpha-1-glycoprotein, serum zinc concentration (SZnC) and retinol. For each micronutrient the relationship between biomarker and dietary intake was investigated by multiple linear regression models accounting for inflammatory biomarkers.

**Results:**

Mean PA for iron, zinc and vitamin A was 0.49, 0.87 and 0.21 among women and 0.61, 0.95 and 0.33 among children, respectively. Prevalence of anemia, corrected low serum ferritin and high sTfR was 37.6%, 4.0% and 77.5% among women and 72.1%, 1.5% and 87.6% among children, respectively. Prevalence of low SZnC and corrected low serum retinol was 39.4% and 12.0% among women and 63.7% and 24.8% among children, respectively. There was a tendency for a positive relationship between vitamin A intakes and serum retinol among women (β = 0.0003, P = 0.06). Otherwise, no link was found between micronutrients biomarkers and intakes.

**Conclusion:**

Our study depicted different images of micronutrient deficiencies when based on dietary intakes or biomarkers results, thus highlighting the need for more suitable biomarkers and more precise measures of absorbable micronutrient intakes at the individual level. It thus points to challenges in the design and evaluation of future biofortification or other food-based interventions in rural areas of Burkina Faso.

## Introduction

Micronutrient deficiencies remain a major public health problem widespread in developing countries [1]. Women and young children are particularly at risk because of their higher needs for vitamins and minerals for pregnancy, lactation or growth, and they may suffer consequences such as higher frequency of illnesses and impairment of physical and mental development [2]. Micronutrient deficiencies thus constitute a major obstacle to the social and economic development of populations [3]. Among strategies to address micronutrient deficiencies, food-based approaches such as dietary diversification, food fortification and biofortification are meant to be sustainable, improving the overall diet quality [4]. In order to implement and evaluate those strategies, it is crucial to estimate the prevalence of micronutrient deficiencies among different population groups in targeted areas, and to assess how much low intakes might contribute to deficiencies.

In Burkina Faso very little is known about levels of vitamin A, iron, and zinc deficiencies. In 2010, according to the Demographic and Health Survey (DHS), the prevalence of anemia was 88% among 6 to 59 months-old children and 49% among 15 to 49 years-old women [5]. It is estimated that around 40–50% of anemia is linked to iron deficiency, globally, but this proportion can vary greatly depending on the prevalence of other factors such as malaria, intestinal parasites and deficiency in vitamin B12 or B9 [6, 7]. Apart from the indirect estimation of iron deficiency through anemia levels, only scarce data is available on other micronutrient deficiencies in Burkina Faso. In rural settings in the Western part of the country, different studies found a decreasing prevalence of zinc deficiency over time: 72.0% in 1999 [8], 62.7% in 2009 [9] and 43.5% in 2012 [10] among children of 6–31, 6–23 and 6–18 months of age, respectively. Vitamin A deficiency seemed to substantially vary by geographical zone of Burkina Faso, ranging from 35 to 85% among children in the *Centre Ouest* [11] and *Centre Nord* [12] regions, respectively, and from 13–17% among adult men and women in urban Ouagadougou, respectively [13], to 64% among women in the *Centre Nord* region [12].

The lack of detailed knowledge about food and nutrients intake and deficiency levels is a substantial obstacle to the design, implementation and evaluation of effective food-based interventions such as biofortification. The present study, a preliminary study of a sorghum biofortification project, was conducted to contribute to fill that knowledge gap in two rural provinces of Burkina Faso. Its objectives were: (i) to provide reliable information on sorghum consumption, micronutrient intakes and micronutrient deficiencies among women of reproductive age and their preschool children, in order to better inform sorghum micronutrient content breeding targets and future interventions; and (ii) to investigate the relationship between biomarkers of status and dietary intakes of iron, zinc and vitamin A among the study subjects in order to better inform the evaluation design of future biofortification interventions.

## Materials and Methods

### Sampling

The survey was cross-sectional and targeted women and preschool children in two rural provinces in western and north-western Burkina Faso: the *Sanguié* province, in the Region of the *Centre-Ouest*; and the *Sourou* province, in the Region of *the Boucle du Mouhoun*. These regions were selected based on a combination of health, agriculture, living conditions and demographics criteria, which included data on sorghum production, household consumption and prevalence of malnutrition. The sampling procedure has been detailed elsewhere [14]. Children aged 36–59 months were selected because at this age they were consuming family dishes and were less likely to be breastfed. Mothers were recruited along with their children for convenience. Precision of the estimates of sorghum consumption was the main outcome used to calculate sample size. Using a coefficient of variation of 0.60 for sorghum intakes, based on previous knowledge about sorghum consumption among women in similar settings, a sample size of 207 subjects was calculated to get a precision of 0.10, with a type I error of 0.05 and accounting for a design effect of 1.5. Allowing for a 10–15% dropout rate led to a sample size rounded to 240 subjects. Previous data indicated that the coefficient of variation for intakes from sorghum-based dishes among young children was lower than 0.60, so the sample size was likely to be sufficient for children. As for micronutrient deficiencies, there was very few previous data available. We therefore considered the worst-case scenario, i.e. prevalence around 50% for at least one biomarker. In such a case, 96 subjects per province would be needed to get a precision of 0.10 with a type I error of 0.05. According to constraints in logistics and financing, we decided to limit the sample size to 90 women and 90 children in each province, which would give the required precision if the prevalence is above 60% or below 40%. The sampling procedure was a multistage cluster selection process of 240 households in each province (5 health areas per province x 6 clusters per area x 8 households per cluster). At the first stage, the health areas were randomly selected proportionally to their population size. At the next stage, 6 clusters (villages) were selected in each health area through the same proportional to size technique. Households were then selected at random from an exhaustive census of all eligible households conducted in each village. In each cluster (n = 30 per province), 3 households were randomly selected to identify mothers and children who underwent biological measurements.

### Dietary data collection and management

Two rounds of dietary data collection were carried out: one in the lean season (July 2010) and one in the post-harvest season (December 2010). Since biomarkers were measured only in the post-harvest season, only second round data is used in the present study. Dietary data was obtained by a standard 24-hour recall multiple-pass method which has been reported in a previous article, as well as detailed characteristics of dietary data management [14]; therefore, only main points are given hereafter. The 24-h recalls used a set of 21 standard recipes, based on the most commonly consumed staple foods, and other recipes were treated as individual household-level recipes of which the quantities of all ingredients were measured, as were final volume of dishes and quantities consumed by mothers and children. A pre-determined preferred method of measurement was decided for each food or ingredient (i.e. direct weighing, standard size, calibrated household measures, photos, prices, units, etc.). At each round, the 24-h recall was repeated on a random subsample of three out of the eight mother-child pairs of each cluster (i.e. 37.5% of repeated measurements) to allow the estimation of the distribution of typical intakes and calculation of the probability of adequacy of nutrient intakes [15]. The random selection of the subsample for repeating the 24-h recall was done independently at each round and independently of the random selection for the biological measurements.

Dietary data were entered in duplicate to limit data-entry errors and foods were transformed in nutrients using CSdietary software (SERPRO and HarvestPlus, Washington DC, 2009). The food composition table (FCT) was a revised version of the one built for a previous study carried out by the Institut de Recherche pour le Développement [16] updated using data from the World Food Dietary Assessment System (University of California at Berkeley International Minilist) and the National Nutrient Database for Standard Reference of the United States Department of Agriculture (Release 20, 2007). The arbitrary threshold method proposed by Willet to identify outliers for energy intakes [17] was adapted in order to account for age, weight and physiological status of women. Regarding children, outliers were identified using the average energy requirement calculated from the formula provided by the Food and Agriculture Organization of the United Nations (FAO) [18] divided by a factor of 0.5 for under-reporters and 4.0 for over-reporters. The probability approach was used to assess usual nutrient intakes and probabilities of adequacy with STATA syntax developed by the Women’s Dietary Diversity Project [19]. This syntax uses Box-Cox transformation to normalize the distribution of energy and nutrients intakes, calculates individual and population means for intakes of each nutrient, as well as within- and between-person variances, to then calculate the best linear unbiased predictor (BLUP) of the usual intake for each nutrient, for each individual. Individual probability of adequacy is then calculated according to the requirement distribution of each nutrient. Estimated Average Requirements (EAR) used were provided by the World Health Organization (WHO) and FAO [20], except for zinc and iron. Zinc adequacy was assessed using the EAR provided by the International Zinc Nutrition Consultative Group when considering a 25% bioavailability [21]. Probability of adequacy for iron was derived from tables I-6 and I-7 from the Institute of Medicine (IOM) [22] assuming a 5% bioavailability.

### Biological data collection and measurement

Two experienced lab technicians were recruited and specially trained to draw blood samples. In each village, the 3/8 surveyed households that were selected were given an appointment at the local health centre, around 8:00 AM, to give blood sample. For both women and children, fasting venous blood samples (10 to 15 mL) were collected by a lab technician assisted by a health centre nurse. Serum was separated by centrifugation at 3000 rpm for 15 minutes within 1h of collection and frozen at -20°C until analyzed. Before and after serum separation, the samples were kept away from the light in cool boxes and brought daily to the base camp established in the main city of the province and then, at the end of sample collection, to the laboratory in Ouagadougou.

Hemoglobin level was measured immediately after blood draw using a Hemocue Hb 201 system (HemoCue AB, Ängelholm, Sweden). Serum retinol concentration was assessed by high performance liquid chromatography method (Agilent HPLC system) at the laboratory of the Institut de Recherches en Sciences de la Santé in Burkina Faso. Serum zinc concentration (SZnC) was determined by atomic absorption spectrometry (Shimadzu AAF-6000) at the laboratory of the Bureau des Mines et de la Géologie du Burkina. The combined Sandwich ELISA technique was used to assess ferritin, C-Reactive Protein (CRP), alpha-1-glycoprotein (AGP), transferrin receptors (sTfR) serum contents [23]. The latter analyses were performed at the VitMin Lab, Willstaett, Germany. Body iron stores (BIS) were calculated using the formula recommended by Cook et al. (i.e. BIS = -[log(sTfR/Ferritin)-2.8229]/0.1207) [24].

Micronutrient deficiencies were determined by the threshold values recommended by the WHO [6, 25, 26]. Anemia was defined as hemoglobin level < 11 g/dL among children and pregnant women and < 12 g/dL among non-pregnant women. Iron deficiency was defined as serum ferritin content < 12 μg/L for children and 15 μg/L for women. Zinc deficiency was defined as serum zinc values below 70 μg/dL. Vitamin A deficiency was defined as serum retinol level < 20 μg/dL. The cut-off values for sTfR, AGP and CRP were levels above 8.3 mg/L, 100 mg/dL and 5 mg/L, respectively. To account for the effect of inflammation on serum ferritin and retinol concentrations, three levels of inflammation were defined: incubation (high CRP level: >5mg/L), early convalescence (high CRP and AGP levels) and late convalescence (high AGP level: >100mg/dL). Correction factors of 0.77 (incubation), 0.53 (early convalescence) and 0.75 (late convalescence) were applied for ferritin [27] and factors of 0.87, 0.76 and 0.89 for incubation, early convalescence and late convalescence, respectively, were applied for retinol [28].

### Socio-demographics characteristics

A household economic scale was computed by multiple correspondence analyses based on the following variables: household assets (cart, animals, moped, mobile phone, and petrol lamp), number of contributors to the overall income of the household, quality of housing (roof, floor and wall materials), access to safe water and latrines. The household food insecurity access scale (HFIAS) was used to gauge food security status of households and was built following recommendations of the Food And Nutrition Technical Assistance project [29].

### Data analysis

Data quality checks, cleaning and statistical analyses were performed separately for women and children using the SAS System version 9.3 (SAS Institute Inc., Cary, NC, USA). All analyses took into account the sampling design by including a random effect for health area in statistical models and results were weighted according to the respective size of the population in each province. Unless specified, the level of significance was set at p-value <0.05 for all analyses.

Biomarkers were checked for extreme values and full distributions were described. Because of their skewness, almost all biomarkers concentrations were log-transformed for both women and children in order to approach normal distribution. The exception was the SZnC which was normally distributed. The transformed variables were then used in statistical models.

For each micronutrient the relationship between biological status, as dependent unadjusted continuous variable, and dietary intake, as independent continuous variable, was studied with multiple linear regression models. AGP and CRP values were introduced as continuous variables into the models to account for inflammation. Age, physiological status and body mass index of women, and age, sex and weight-for-age z-score of children were systematically accounted for. To explore modifier effects of household or individual socio-economic and demographic characteristics on this relationship, a set of potential factors was identified through separate regression analyses on the biomarkers and on the dietary intakes. Factors linked to both variables with a type I error below 0.20 were included in the final model. Interactions between these factors and dietary intakes were systematically tested. The same procedure was reproduced to examine the odds of being deficient for each micronutrient, according to dietary intake, using multiple logistic regression models.

### Ethics

The study protocol was approved by the National Ethic Committee of the Ministry of Health in Burkina Faso (decision number 2010–52 on July 7^th^, 2010). Written informed consent was obtained from all mothers and/or heads of households, for both the mother herself and for her child, on a separate form. All cases of anemia detected during blood sampling received iron and folic acid tablets. The study was conducted in accordance with the ethical guidelines of the 2008 Declaration of Helsinki.

## Results

### Description of the sample

The final sample included 478 households with an average of 8.6 people per household (Table 1). Households in the *Sourou* province appeared to be better-off than those in the *Sanguié* province both in terms of housing quality (wall, floor, roof, latrine, etc.) and ownership of assets (lamp, radio, plough with animals to draw it, livestock, etc.). This was reflected through the household economic scale: 51.0% of households in the *Sanguié* were in the lowest tertile of this economic scale while the same proportion was in the highest tertile in the *Sourou*. According to the HFIAS score, less than half of households appeared to be food secure in each province. The sample included 57.6% of lactating non-pregnant women (hereafter simply referred to as ‘lactating women’), 31.2% of non-pregnant non-lactating women and 11.2% of pregnant women. Women were aged 31 years old on average, 13.4% of them were underweight and 4.5% overweight. Children were 50.1 months old on average. Of children, 29.6% were found to be stunted and 1.8% to be wasted.

**Table 1:** Socio-economic and demographic characteristics of the sample.

	*Sanguié*	*Sourou*		Total
Household	% (n = 239)	% (n = 239)	P-value^1^	% (n = 478)
Household economic scale				
*Poor*	51.0	15.5	0.002	35.9
*Medium*	33.1	33.5		33.2
*Rich*	15.9	51.0		30.9
Household food insecurity access scale				
*Food secure*	47.7	43.5	0.66	45.9
*Food insecure*	52.3	56.5		54.1
Women	% (n = 228)	% (n = 227)	P-value^1^	% (n = 455)
Age				
*< 30*	43.4	42.3	0.74	42.9
*≥ 30*	56.6	57.7		57.1
Physiological status				
*Non-pregnant*, *non-lactating*	28.5	34.8	0.23	31.2
*Pregnant*	10.1	12.8		11.2
*Lactating*	61.4	52.4		57.6
Body Mass Index				
*Underweight (BMI < 18*.*5)*	14.1	12.6	0.84	13.4
*Normal (18*.*5* ≤ *BMI < 25)*	81.1	83.4		82.0
*Overweight and more (BMI* ≥ *25)*	4.8	4.0		4.5
Children	% (n = 226)	% (n = 222)	P-value^1^	% (n = 448)
Age				
*36–47 months*	43.8	36.9	0.048	40.9
*48–59 months*	56.2	63.1		59.1
Gender				
*Male*	50.4	48.2	0.65	49.5
*Female*	49.6	51.8		50.5
Weight-for-age < -2 Z-scores				
*Yes*	12.2	15.6	0.48	13.6
*No*	87.8	84.4		86.4
Height-for-age < -2 Z-scores				
*yes*	28.5	31.1	0.61	29.6
*no*	71.5	68.9		70.4
Weight-for-height < -2 Z-scores				
*yes*	0.5	3.8	0.007	1.8
*no*	99.5	96.2		98.2

^1^ Chi-square test

### Micronutrient intakes and biomarkers

Twenty-four women (5.0%) and 32 children (6.7%) were identified as over- or under-reporters, so dietary data was finally available for 455 women and 448 children. Depending on the biomarker considered, sample size for biomarkers varied from 150 to 166 women and from to 132 to 155 children, corresponding to 83–92% and 73–86% of the selected subsample of women and children, respectively.

Probabilities of adequacy (PA) for the three micronutrients tended to be higher for children than for women and higher in the *Sourou* than in the *Sanguié* (Table 2). PAs for zinc were notably high, ranging from 0.83 to 0.96, while PAs for iron were middling, ranging from 0.46 to 0.67, and PAs for vitamin A were low, ranging from 0.17 to 0.43.

**Table 2:** Micronutrient usual intakes and probability of adequacy among women and children by province.

	EAR ^1^	N	Mean	(Min–Max)	Median	(Q1–Q3)	PA (mean)	95% CI
Women								
*Sanguié*								
Iron, mg ^2^	29.1; 49.9; 23.4	228	26.1	(6.7–67.2)	24.6	(20–30.8)	0.46	[0.44, 0.49]
Zinc, mg ^3^	7; 10; 8	228	12.8	(2.9–32.1)	12.5	(10.1–15)	0.90	[0.84, 0.96]
Vitamin A, μg RE	270; 370; 450	228	212	(2–1736)	138	(69–295)	0.17	[0.03, 0.32]
*Sourou*								
Iron, mg ^2^	29.1; 49.9; 23.4	227	29.4	(9.2–64.9)	28.5	(22.6–34.2)	0.53	[0.42, 0.63]
Zinc, mg ^3^	7; 10; 8	227	11.2	(3.4–23.4)	10.9	(8.9–13.1)	0.83	[0.76, 0.90]
Vitamin A, μg RE	270; 370; 450	227	296	(11–1560)	237	(169–356)	0.27	[0.21, 0.33]
Children								
*Sanguié*								
Iron, mg ^2^	10.8; 14.8	226	15.0	(1.9–32.8)	14.5	(11.4–17.7)	0.57	[0.52, 0.62]
Zinc, mg ^3^	2; 4	226	6.9	(1.6–15.7)	6.6	(5.6–8.2)	0.96	[0.91, 1.00]
Vitamin A, μg RE	200; 200	226	151	(0–920)	93	(46–206)	0.26	[0.10, 0.42]
*Sourou*								
Iron, mg ^2^	10.8; 14.8	222	18.1	(5.0–50.3)	17.3	(13.5–21.2)	0.67	[0.59, 0.75]
Zinc, mg ^3^	2; 4	222	6.4	(1.9–13.0)	6.3	(5.2–7.5)	0.94	[0.87, 1.00]
Vitamin A, μg RE	200; 200	222	231	(0–1614)	176	(118–271)	0.43	[0.36, 0.51]

^1^ Estimated Average Requirement (EAR) are given for non-pregnant non-lactating women; pregnant women; lactating women, respectively; and for children younger and older than 48 months-old, respectively.

^2^ EAR are given for a 5% iron absorption level.

^3^ EAR are given for a 25% zinc absorption level with the exception of lactating women for which a 35% absorption level is considered.

Inflammation was fairly common, especially among children, and more pronounced for women in the *Sanguié* than in the *Sourou* (Table 3). In total, there were 17.1% and 30.7% of women and 10.4% and 8.6% of children without inflammation in the *Sanguié* and in the *Sourou*, respectively. Anemia was frequent among children and, to a lesser extent, among women. No difference occurred between provinces for children, while women in the *Sourou* were more likely to be anemic than women in the *Sanguié*. Iron deficiency, measured by corrected low serum ferritin, was very rare. However, iron deficiency measured by body orn stores was a bit higher, particularly in the *Sourou*. In addition, a huge percentage of subjects had high concentration of serum transferrin receptors, indicating tissue iron deficiency. Zinc deficiency affected almost three quarters of children and one quarter of women in the *Sanguié* and around half of both children and women in the *Sourou*. A similar pattern was found for corrected vitamin A deficiency: while it affected 30.6% of children and only 6.0% of women in the *Sanguié*, it was around 18% for both children and women in the *Sourou*.

**Table 3:** Biological status and percentage of deficiency among women and children by province.

	Cut-off value	N	Mean or %	(Min–Max)	Median	(Q1–Q3)	% above or under cut-off value	95% CI
Women from *Sanguié*								
AGP, mg/dL	> 100	76	141.5	(59.0–300.0)	134.0	(108.0–163.0)	80.3	[64.8, 95.7]
CRP, mg/L	> 5	76	5.5	(0.0–56.6)	2.0	(0.8–3.9)	21.1	[13.8, 28.3]
Inflammation status (%) ^1^								
No inflammation	-	76	17.1	-	-	-	-	[6.4, 27.8]
Incubation	-	76	2.6	-	-	-	-	[0.0, 9.7]
Early convalescence	-	76	18.4	-	-	-	-	[6.6, 30.3]
Late convalescence	-	76	61.8	-	-	-	-	[48.4, 75.3]
Ferritin, μg/L	< 15	76	119.6	(13.6–354.2)	104.5	(56.2–157.1)	2.6	[0.0, 7.2]
Ferritin corrected, μg/L ^2^	< 15	76	89.0	(10.3–265.6)	78.4	(39.9–118.7)	2.6	[0.0, 7.2]
sTfR, mg/L	> 8.3	76	12.8	(5.8–30.0)	12.4	(9.2–15.6)	78.9	[53.4, 100]
BIS, mg/kg ^3^	< 0	76	4.9	(-5.1–11.0)	5.0	(2.5–7.4)	3.9	[0.0, 11.5]
Hemoglobin, g/dL	< 12–11	85	12.5	(8.6–15.9)	12.4	(11.5–13.5)	29.4	[10.1, 48.7]
Zinc, μg/dL	< 70	82	81.0	(52.0–115.5)	80.0	(68.6–91.8)	25.6	[0.0, 60.1]
Retinol, μg/dL	< 20	83	35.9	(6.5–71.5)	35.4	(27.6–42.9)	7.2	[0.0, 15.7]
Retinol corrected, μg/dL ^4^	< 20	83	40.6	(6.5–83.4)	39.0	(30.4–47.7)	6.0	[0.0, 15.4]
Women from *Sourou*								
AGP, mg/dL	> 100	75	118.5	(42.0–238.0)	114.5	(88.8–141.5)	68.0	[57.8, 78.2]
CRP, mg/L	> 5	75	3.9	(0.0–37.0)	2.2	(0.6–3.7)	13.3	[4.3, 22.4]
Inflammation status (%) ^1^								
No inflammation	-	75	30.7	-	-	-	-	[20.7, 40.6]
Incubation	-	75	1.3	-	-	-	-	[0.0, 5.1]
Early convalescence	-	75	12.0	-	-	-	-	[2.3, 21.7]
Late convalescence	-	75	56.0	-	-	-	-	[45.9, 66.1]
Ferritin, μg/L	< 15	75	102.0	(6.4–339.8)	84.1	(42.0–147.5)	2.7	[0.0, 7.3]
Ferritin corrected, μg/L ^2^	< 15	75	79.4	(5.8–254.9)	71.7	(32.9–105.5)	5.3	[0.0, 12.5]
sTfR, mg/L	> 8.3	75	12.8	(4.1–33.0)	12.1	(8.3–15.1)	76.0	[65.1, 86.9]
BIS, mg/kg ^3^	< 0	75	4.4	(-7.7–12.2)	5.0	(1.8–7.3)	13.3	[4.7, 22.0]
Hemoglobin, g/dL	< 12–11	85	11.9	(3.5–18.8)	11.9	(10.9–13.1)	45.9	[30.6, 61.1]
Zinc, μg/dL	< 70	83	70.5	(33.8–129.7)	68.3	(57.7–80.1)	53.0	[17.8, 88.3]
Retinol, μg/dL	< 20	84	26.6	(7.7–74.4)	24.6	(20.6–31.6)	20.2	[7.0, 33.4]
Retinol corrected, μg/dL ^4^	< 20	84	29.1	(8.7–74.4)	27.0	(22.2–34.0)	17.9	[5.5, 30.2]
Children from *Sanguié*								
AGP, mg/dL	> 100	67	172.2	(53.0–300.0)	159.0	(130.4–208.5)	89.6	[80.6, 98.5]
CRP, mg/L	> 5	67	15.2	(0.3–63.4)	4.9	(2.8–20.8)	50.7	[24.7, 76.7]
Inflammation status (%) ^1^								
No inflammation	-	67	10.4	-	-	-	-	[1.5, 19.4]
Incubation	-	67	0.0	-	-	-	-	[0.0, 0.0]
Early convalescence	-	67	50.7	-	-	-	-	[24.7, 76.7]
Late convalescence	-	67	38.8	-	-	-	-	[17.2, 60.4]
Ferritin, μg/L	< 12	67	129.2	(18.7–427.9)	105.0	(63–169.1)	0.0	[0.0, 0.0]
Ferritin corrected, μg/L ^2^	< 12	67	79.7	(12.9–226.8)	64.7	(41.9–103.3)	0.0	[0.0, 0.0]
sTfR, mg/L	> 8.3	67	13.5	(2.8–27.4)	13.3	(9.7–16.7)	83.6	[71.8, 95.4]
BIS, mg/kg ^3^	< 0	67	4.5	(-2.5–10.2)	4.7	(3.0–6.4)	6.0	[0.0, 12.3]
Hemoglobin, g/dL	< 11	82	10.1	(6.5–12.8)	10.0	(9–11)	72.0	[63.7, 80.2]
Zinc, μg/dL	< 70	83	61.2	(25.4–101.4)	60.0	(51.3–71.2)	73.5	[38.7, 100]
Retinol, μg/dL	< 20	85	25.0	(4.7–74.9)	22.5	(15.9–29.8)	34.1	[3.1, 65.1]
Retinol corrected, μg/dL ^4^	< 20	85	29.0	(4.7–74.9)	24.7	(18.8–37.1)	30.6	[1.4, 59.7]
Children from *Sourou*								
AGP, mg/dL	> 100	70	174.8	(71.0–300.0)	170.0	(131.0–212.0)	90.0	[82.0, 98.0]
CRP, mg/L	> 5	70	15.6	(0–62.8)	4.4	(1.3–26.1)	45.7	[23.8, 67.7]
Inflammation status (%) ^1^								
No inflammation	-	70	8.6	-	-	-	-	[2.9, 14.2]
Incubation	-	70	1.4	-	-	-	-	[0.0, 5.2]
Early convalescence	-	70	44.3	-	-	-	-	[24.9, 63.7]
Late convalescence	-	70	45.7	-	-	-	-	[23.0, 68.5]
Ferritin, μg/L	< 12	70	134.4	(6.2–405.4)	119.6	(63.7–193.2)	1.4	[0.0, 5.2]
Ferritin corrected, μg/L ^2^	< 12	70	85.7	(6.2–214.8)	75.5	(42.3–122.7)	2.9	[0.0, 7.6]
sTfR, mg/L	> 8.3	70	16.2	(6.7–26.7)	16.3	(12.7–19.7)	91.4	[79.7, 100]
BIS, mg/kg ^3^	< 0	70	3.8	(-6.5–8.9)	4.4	(1.7–6.6)	12.9	[0.0, 32.2]
Hemoglobin, g/dL	< 11	83	9.6	(3.6–13.2)	9.8	(8.5–11.0)	72.3	[63.8, 80.8]
Zinc, μg/dL	< 70	74	69.7	(17.5–110.4)	67.0	(56.0–87.1)	52.7	[26.6, 78.8]
Retinol, μg/dL	< 20	76	26.0	(5.1–85.7)	23.4	(17.7–29.4)	34.2	[25.5, 42.9]
Retinol corrected, μg/dL ^4^	< 20	76	30.0	(6.7–96.3)	27.6	(21.1–35.2)	18.4	[13.0, 23.8]

^1^ No inflammation: AGP < 1.0 g/L and CRP < 5.0 mg/L; incubation: AGP < 1.0 g/L and CRP ≥ 5.0 mg/L; early convalescence: AGP ≥ 1.0 g/L and CRP ≥ 5.0 mg/L; late convalescence: AGP ≥ 1.0 g/L and CRP < 5.0 mg/L.

^2^ Correction factors are applied according to the level of inflammation based on AGP and CRP concentrations: serum ferritin is multiplied by 0.77 during incubation, 0.53 during early convalescence and 0.75 during late convalescence.

^3^ Body Iron Stores = -[log(sTfR/Ferritin)-2.8229]/0.1207, using corrected value of Ferritin.

^4^ Correction factors are applied according to the level of inflammation based on AGP and CRP concentrations: serum retinol is divided by 0.87 during incubation, 0.76 during early convalescence and 0.89 during late convalescence.

### Relationships between intakes and biomarkers

Coefficients of determination (R-square) of the multiple linear regression models with biomarkers as dependent variables ranged from 0.10 to 0.38 (Table 4). Among all socio-demographic and economic factors tested, including age, anthropometry, women’s physiological status, child gender, household economic status and food insecurity, only the province had a significant effect on biomarkers (with a p<0.20 threshold) and was therefore included in the models. There was no significant interaction between intakes and cofactors. Among women, there was a tendency for a positive relationship between vitamin A intakes and serum retinol: an intake of 100 μg RE of vitamin A was associated with a slight increase of 0.03 μg/dL of serum retinol (p = 0.06); otherwise, the effects of micronutrient intakes on biomarker values were not significant. Using multiple logistic regression models we couldn’t find any significant risk of being deficient according to dietary intake, for any micronutrient (results not shown).

**Table 4:** Relationships between biomarkers and micronutrient intakes, adjusted on cofactors including inflammation markers (multivariate linear regression models).

	Ferritin μg/L ^1^			sTfR mg/L ^1^			Zinc μg/dL			Retinol μg/dL ^1^		
	Coeff.	SD	P-value^2^	Coeff.	SD	P-value^2^	Coeff.	SD	P-value^2^	Coeff.	SD	P-value^2^
Women												
Intake	-0.006	0.004	0.14	-0.002	0.004	0.74	-0.170	0.470	0.73	0.0003	0.0001	0.06
CRP	0.003	0.006	0.68	-0.008	0.003	0.03	0.230	0.086	0.027	-0.003	0.002	0.241
AGP	0.004	0.002	0.04	0.004	0.001	0.0001	-0.022	0.028	0.458	0.001	0.001	0.136
Age (years)	-0.016	0.012	0.23	0.003	0.004	0.41	0.114	0.146	0.46	0.001	0.006	0.92
Physiological status												
Pregnant	0.047	0.137	0.77	-0.090	0.059	0.02	0.209	2.385	0.08	0.083	0.082	0.61
NPNL	0.129	0.179	0.77	-0.199	0.097	0.02	-10.544	3.990	0.08	-0.033	0.106	0.61
Lactating ^3^	-	-	-	-	-	-	-	-		-	-	-
Body Mass Index	-0.019	0.021	0.40	0.007	0.019	0.70	0.470	0.595	0.45	-0.011	0.025	0.66
Province												
*Sanguié*	0.093	0.122	0.47	-0.068	0.060	0.29	12.217	5.674	0.06	0.292	0.091	0.01
*Sourou* ^3^	-	-	-	-	-	-	-	-	-	-	-	-
N	150			150			144			149		
R^2^	0.10			0.30			0.20			0.18		
P-value (Model)	0.00001			0.0006			0.00001			0.0001		
Children												
Intake	0.011	0.010	0.29	0.0001	0.005	0.93	-0.828	0.775	0.32	-0.0002	0.0001	0.23
CRP	0.015	0.002	0.0001	-0.003	0.001	0.05	-0.033	0.047	0.497	-0.007	0.003	0.046
AGP	0.004	0.001	0.004	0.004	0.001	0.0001	-0.068	0.026	0.033	0.001	0.0001	0.153
Age (month)	0.001	0.009	0.95	0.009	0.004	0.07	0.063	0.173	0.73	-0.001	0.010	0.91
Gender												
Male	-0.112	0.054	0.07	-0.018	0.066	0.79	2.653	3.549	0.48	-0.180	0.083	0.06
Female ^3^	-	-	-	-	-	-	-	-	-	-	-	-
WAZ ^4^	0.062	0.065	0.37	-0.027	0.038	0.50	-1.884	0.789	0.04	0.025	0.046	0.60
Province												
*Sanguié*	0.038	0.134	0.79	-0.179	0.081	0.06	-9.117	2.992	0.02	-0.007	0.110	0.95
*Sourou* ^3^	-	-	—	-	-	-	-	-	-	-	-	-
N	131			131			126			128		
R^2^	0.38			0.40			0.20			0.13		
P-value (Model)	0.00001			0.00001			0.00001			0.0001		

^1^ Log-transformed variable

^*2*^ Wald Chi-square test

^*3*^ Reference category (no coefficient, no SD)

^*4*^ Weight-for-age (Z-score)

## Discussion

In this sample of rural women and children in Burkina Faso the probability of adequacy of dietary intakes slightly varied according to the province and was quite high for zinc (0.83–0.96), low for vitamin A (0.17–0.43) and somewhat moderate for iron (0.46–0.67). However, when assessed by biomarker values, the deficiency levels were rather high for zinc (25.6–73.5%) and only moderate for vitamin A (6.0–30.6%). Iron deficiency according to corrected ferritin values appeared very low (0–2.7%), despite a high rate of anemia (29.4–72.3%). But the high level of sTfR concentrations that we observed (76.0–91.4%) may suggest that iron deficiency is in fact much more prevalent than indicated by the rates of low serum ferritin. No link was found between dietary intakes and micronutrient biomarkers, except a borderline relationship for retinol among women. No socio-economic or demographic characteristics were identified as modifying or confounding factors for these relationships or lack thereof.

Low dietary intakes are deemed to be the primary cause of micronutrient deficiencies in developing countries, so the lack of relationship between biomarkers and dietary intake seems counter-intuitive. Such discrepancies have nevertheless been repeatedly described in the literature, notably for iron [30–33] and zinc [34–37], but less clearly for vitamin A [37–41]. The lack of cohesion between micronutrient intake and status is unfortunately a limitation of the nutritional science at the moment. There is still no cohesive model that provides a reliable and consistent explanation of the relationship between intake and status because of limitations on both sides. The closest to a cohesive model at the moment are regression models between those two variables (intake and status) from meta analyses using pooled randomized controlled trials data with supplementation or fortification [42–44]. However, if we take the example of iron, supplemental doses in those studies were typically high, representing several times the amount of iron intake observed in our study. These studies also typically take into account factors that could affect status such as inflammation, as we did, but also treat hookworm infestation before the intervention. In observational real life conditions studies such as ours, this is very rarely the case.

Broadly speaking, dietary intakes that we measured were in the range of those found by other studies in Burkina Faso among women’s [16, 45] or children [11, 46]. The small discrepancies came from slightly different methods, contexts or seasons. In other contexts in Africa, among children, vitamin A intakes were found to be in the same range as ours, but zinc and iron intakes were lower [47], probably because in our sample sorghum, which has high iron and zinc contents, was the main staple food in diet [48].

In our study, rates of anemia were lower than those observed in the rural sample of the latest DHS in the country [5], both among women (35.7 vs 51.1%) and children (72.4 vs 89.9%). To some extent, this could be linked to the higher consumption of iron we highlighted above, even if its bioavailability might be overestimated as we will discuss later. As compared to studies from other African contexts [49, 50], in our sample anemia was in the same range but prevalence of low ferritin was lower and level of sTfR concentrations was higher; and the rate of inflammation was notably high for both women and children in our study. Another explanation for the high ferritin values that we observed could be the contamination of foods with iron rich soil, a phenomenon that has been already highlighted in this particular context [51]. Indeed, our study also revealed that iron content of meals made out of sorghum grains was 5 to 6 times higher than iron content of the grains themselves [52]. Nevertheless, iron deficiency is likely to be underestimated when gauged by serum ferritin, even after adjustment, because of the high level of inflammation in our sample. It is indeed a bit higher if we consider body iron stores and the high levels of sTfR indicated large tissue iron deficiency. Indeed, sTfR concentration usually increases in case of hemolytic anemia [53], which can be induced by parasite infestations such as hookworm or malaria [7], but is deemed not to rise in presence of inflammation [25] though this is still debated [54–56]. In any case, the high rate of inflammation that we observed leads to the hypothesis that in our sample anemia in women and children was at least partially of hemolytic origin. Unfortunately parasites were not looked for at the time of data collection but we were able to assess the level of malaria infestation by testing stored serum samples from one of the two provinces. Malaria antibodies were found for 63.3% of women and 15.6% of children, which indicate a holoendemic area and is a likely explanation for the apparent discrepancy between ferritin and sTfR concentrations.

Another consideration for iron deficiency is bioavailability of dietary iron. In the present study a 5% bioavailability was assumed, but it is well known that many factors play an important role to enhance or inhibit iron absorption. In our study iron dietary intakes mainly came from high phytate content foods such as sorghum and other grains [14]; therefore even 5% bioavailability for iron may be overestimated. In addition, not having been able to account for inhibitors or enhancers of iron absorption at the individual level is likely to have obscured the relationship between iron intakes and iron deficiency status.

The prevalence of zinc deficiency was on average 38.6% among women and 64.3% among children in our study, though some differences between the two provinces were noted. Studies on children in rural Burkina Faso quoted in the introduction revealed prevalence levels similar to those in our sample, ranging from 43.5% to 72.0% [8–10]. In Cameroon, among women and children [36], and among Ghanaian women [57], zinc deficiency was found to be much more important than in our study. But the most striking of our results was the high prevalence of zinc deficiency while intakes were very high, for both women and children. At the population level, it is commonly thought that levels of zinc intakes and SZnC should correlate [58]. In Cameroon, however, examining differences between regions, SZnC appeared to be negatively related to dietary zinc intake [36]. Such discrepancies might well come from the variability of intakes measurement as well as differences in zinc bioavailability [59]. Indeed, as for iron, it would have been useful to calculate intakes of absorbable zinc if we had been able to measure phytate intakes. In addition, as far as we know there are no published correction factors for adjusting plasma SZnC for inflammation. This should not affect the assessment of the relationship between diet and biomarkers, but it would allow better comparison of the proportion of low values using one or the other.

Vitamin A deficiency prevalence in our study was on average 13.3% among women and 33.9% among children. As for zinc, some differences between the two provinces were noted. As stated in the introduction, other studies in Burkina Faso revealed varied levels of vitamin A deficiency, depending on setting and season [11–13]. Vitamin A deficiency figures from Côte d’Ivoire [60] and Cameroon [49] were in the same range as ours, but those studies used the retinol binding protein indicator. Our regression analysis found a relationship between serum retinol and vitamin A intakes, though not very strong and among women only. We failed to find in the literature any similar study in developing countries and/or the studies we found estimated vitamin A intakes in a quite different way [38, 39, 41, 61]. Among children, a study in Zambia found a relationship between plasma retinol and vitamin A intake, but only when using a 26:1 retinol equivalence for provitamin A from green and yellow vegetables [40]. This study also underscored the still unclear role that inflammation could play in the association between vitamin A intake and deficiency [40]. Indeed, serum retinol reflects liver vitamin A stores only if they are < 0.7 or > 1.05 μmol/g liver [62]; otherwise, serum retinol is biologically controlled, making it difficult to assess the individual vitamin A status [26].

Our study acknowledges some strengths and weaknesses. Among the strengths was the extensive knowledge of the investigators of dietary habits in Burkina Faso and their experience in conducting dietary surveys in the country. The study design reduced sampling errors and improved the representativeness of the sample. However, since the study area was selected because of its high levels of sorghum production and consumption, the external validity of our results remains limited even though general living conditions are similar to what is observed in many parts of the rural Sahel. Among weaknesses, though much attention was paid to update our FCT, some details that could have been valuable for the analysis were not included. For example, it would have been better to distinguish between de-hulled and not de-hulled grains or between two varieties of a same grain (e.g. red or white sorghum) because their nutritional contents, in phytate particularly, are different. We could also have calculated alternative vitamin A RAE intakes by using a retinol equivalency of 26:1 for green vegetables as this was shown to improve relationship between intakes and retinol concentration [40]. Indeed, in our sample approximately two thirds of the vitamin A in the diet came from dark green leafy vegetables. Building a more complete FCT and collecting the corresponding detailed information in the 24h-recall can be extremely complex but may be worth the effort when results are used at the individual level. Although we used the best practice methodology to assess probabilities of adequacy, the 24h-recall may also not be the best instrument to estimate long-term intakes [63] and thus to assess their relationships with biomarkers. As we had collected dietary data on same individuals a few months earlier we calculated “long-term usual intakes” over the 2 rounds. The main difference was that long-term usual iron intakes appeared lower, mainly in the *Sourou* province and particularly for children (S1 Table). In the regression analysis the use of those “long-term usual intakes” didn’t improve statistical performance (R-square values were roughly the same) nor changed our conclusions (results not shown). Another drawback to study the relationship between micronutrient intakes and biomarkers was the rather small size of our sample. As stated earlier, the study was originally designed to assess the level of dietary intakes and micronutrient deficiencies, and sample size was calibrated accordingly but not to gauge the relationship between the two.

## Conclusion

This study highlights that the actual association between dietary intakes of iron, zinc and vitamin A and the corresponding biomarkers in individuals is not systematic and difficult to demonstrate. There are still numerous challenges to elucidate those relationships [4]. On the one hand, there is a need of more suitable biomarkers at the individual level and to some agreement about the correction factors that should be applied in presence of inflammation, malaria, or other parasitic diseases. On the other hand, more complete dietary studies are needed: either carried out over longer time periods and/or with more sophisticated analysis to estimate absorbable intakes. Such studies may be very complex and lengthy before establishing firm recommendations. In addition of, and by comparison to studies like ours, relationships between micronutrient intakes and biomarkers should be more frequently studied in healthy populations to better understand how they vary by context. In spite of its limitations, our study provided abundant information about either micronutrient intake or status which was lacking in Burkina Faso. At the same time, it challenged the view of measuring biomarkers as a way to assess the impact of program providing better or higher quality foods and provided a better base for designing and evaluating future interventions and strategies that might help to fight micronutrient deficiencies.

## Supporting Information

S1 Table Micronutrient long-term usual intakes and probability of adequacy among women and children by province. (DOCX)
