# Supplementary material for: Biological Status and Dietary Intakes of Iron, Zinc and Vitamin A among Women and Preschool Children in Rural Burkina Faso
Source: PLoS One. 2016 Mar 18;11(3):e0146810. doi: 10.1371/journal.pone.0146810 (PMC4798773; doi:10.1371/journal.pone.0146810)
Supplement: S1 Table — (DOCX) [file pone.0146810.s001.docx]

**S1 Table. Micronutrient long-term^1^ usual intakes and probability of adequacy among women and children by province**

|  | EAR ^2^ | N | Mean | (Min - Max) | Median | (Q1 - Q3) | PA (mean) | 95% CI |
| --- | --- | --- | --- | --- | --- | --- | --- | --- |
| Women |  |  |  |  |  |  |  |  |
| *Sanguié* |  |  |  |  |  |  |  |  |
| Iron, mg ^3^ | 29.1 ; 49.9 ; 23.4 | 228 | 24.2 | (9.7 - 43.8) | 23.4 | (19.5 - 28.2) | 0.42 | [0.37, 0.47] |
| Zinc, mg ^4^ | 7 ; 10 ; 8 | 228 | 12.2 | (5.3 - 22.3) | 12.1 | (10.2 - 14.0) | 0.92 | [0.89, 0.96] |
| Vitamin A, µg RE | 270 ; 370 ; 450 | 228 | 229 | (10 - 1192) | 184 | (110 - 293) | 0,18 | [0.02, 0.33] |
| *Sourou* |  |  |  |  |  |  |  |  |
| Iron, mg ^3^ | 29.1 ; 49.9 ; 23.4 | 227 | 24.3 | (9.6 - 49.1) | 23.8 | (19.6 - 28.0) | 0.39 | [0.24, 0.54] |
| Zinc, mg ^4^ | 7 ; 10 ; 8 | 227 | 11.0 | (4.2 - 26.3) | 10.7 | (8.8 - 12.7) | 0.84 | [0.73, 0.95] |
| Vitamin A, µg RE | 270 ; 370 ; 450 | 227 | 285 | (31 - 1066) | 247 | (187 - 341) | 0.26 | [0.10, 0.43] |
| Children |  |  |  |  |  |  |  |  |
| *Sanguié* |  |  |  |  |  |  |  |  |
| Iron, mg ^3^ | 10.8 ; 14.8 | 226 | 14.1 | (4.5 - 29.7) | 13,7 | (11.6 - 16.4) | 0.55 | [0.5, 0.6] |
| Zinc, mg ^4^ | 2 ; 4 | 226 | 6.9 | (3.0 - 14.1) | 6,8 | (5.8 - 7.8) | 0.99 | [0.96, 1.01] |
| Vitamin A, µg RE | 200 ; 200 | 226 | 162 | (8 - 723) | 128 | (82 - 196) | 0.27 | [0.11, 0.44] |
| *Sourou* |  |  |  |  |  |  |  |  |
| Iron, mg ^3^ | 10.8 ; 14.8 | 222 | 14.7 | (5.4 - 32.9) | 14.3 | (11.3 - 17.7) | 0.55 | [0.46, 0.64] |
| Zinc, mg ^4^ | 2 ; 4 | 222 | 6.3 | (2.0 - 12.4) | 6.2 | (5.2 - 7.3) | 0.95 | [0.90, 1.01] |
| Vitamin A, µg RE | 200 ; 200 | 222 | 195 | (6 - 580) | 162 | (122 - 243) | 0.39 | [0.31 - 0.46] |

^1^ Long-term usual intakes are calculated over 2 rounds of data collection which took place a few months apart, each round with a 3/8 repetition of the 24-hour recalls;

^2^ Estimated Average Requirement (EAR) are given for non-pregnant non-lactating women, pregnant women and lactating women respectively; and for children younger and older than 48 months-old respectively.

^3^  EAR are given for a 5% iron absorption level.

^4^  EAR are given for a 25% zinc absorption level with the exception of lactating women for which a 35% absorption level is considered.
